# Effectiveness of a support intervention for family caregivers and stroke survivors^*^

**DOI:** 10.1590/1518-8345.4991.3482

**Published:** 2021-09-03

**Authors:** Jaine Kareny da Silva, Rita Narriman Silva de Oliveira Boery

**Affiliations:** 1 Universidade do Estado da Bahia, Departamento de Educação - Campus XII, Guanambi, BA, Brazil.; 2 Scholarship holder at the Programa de Apoio à Capacitação Docente e de Técnicos Administrativos (Bolsa PAC-DT)/ Universidade do Estado da Bahia, Brazil.; 3 Universidade Estadual do Sudoeste da Bahia, Departamento de Saúde, Jequié, BA, Brazil.

**Keywords:** Evidence-Based Nursing, Caregivers, Family, Stroke, Program, Social Support, Enfermagem Baseada em Evidências, Cuidadores, Família, Acidente Vascular Cerebral, Programa, Apoio Social, Enfermería Basada en la Evidencia, Cuidadores, Familia, Accidente Cerebrovascular, Programa, Apoyo Social

## Abstract

**Objective:**

to analyze the effectiveness of a support intervention on the burden and stress of family caregivers and on the stroke survivors’ independence level, compared to the Control Group.

**Method:**

a quasi-experimental study conducted with 37 participants (Intervention Group, n=20; and Control Group, n=17). The intervention lasted 8 months. The outcomes of the caregivers (burden and stress) and of the survivors (independence level) were measured by the Zarit, Perceived Stress and Katz scales, at the following moments: pre-intervention, the fourth month of the intervention and post-intervention. The differences of these outcomes between groups and intra-group and the effect size were calculated using the Mann-Whitney and Friedman tests (Bonferroni adjustment by Wilcoxon) and the Kendall’s W coefficient.

**Results:**

the Intervention Group reduced burden (p=0.039) and stress (p=0.009), mainly, after 8 months of intervention, which was not observed in the Control Group. The independence level did not change between the groups or moments (p>0.05). The intervention presented moderate effect size (p=0.45 and p=0.54).

**Conclusion:**

the intervention was effective to reduce the burden and stress of family caregivers, but did not alter the stroke survivors’ independence level, when compared to the Control Group.

## Abstract (pt)

**Objetivo:**

analisar a efetividade de uma intervenção de apoio na sobrecarga e no estresse de cuidadores familiares e no grau de independência dos sobreviventes de Acidente Vascular Cerebral, em comparação ao Grupo Controle.

**Método:**

estudo quase-experimental realizado com 37 participantes (Grupo Intervenção n=20 e Grupo Controle n=17). A intervenção durou 8 meses. Os desfechos dos cuidadores (sobrecarga e estresse) e dos sobreviventes (grau de independência) foram mensurados pelas escalas de Zarit, Estresse Percebido e Katz, nos momentos: pré-intervenção, quarto mês da intervenção e pós-intervenção. As diferenças desses desfechos intergrupos e intragrupo e o tamanho do efeito foram calculados pelos testes de Mann-Whitney e de Friedman (ajuste de Bonferroni pelo Wilcoxon) e o coeficiente de Kendall W.

**Resultados:**

o Grupo Intervenção reduziu a sobrecarga (p=0,039) e o estresse (p=0,009), principalmente, após 8 meses de intervenção, o que não foi observado no Grupo Controle. O grau de independência não se alterou entre os grupos ou momentos (p>0,05). A intervenção apresentou tamanho de efeito moderado (p=0,45 e p=0,54).

**Conclusão:**

A intervenção foi efetiva para reduzir a sobrecarga e o estresse dos cuidadores familiares, mas não alterou o grau de independência dos sobreviventes de Acidente Vascular Cerebral, quando comparada ao Grupo Controle.

## Abstract (es)

**Objetivo:**

analizar la efectividad de una intervención de apoyo sobre la sobrecarga y el estrés de los cuidadores familiares y sobre el grado de independencia de los sobrevivientes de accidente cerebrovascular, en comparación con el Grupo de Control.

**Método:**

estudio cuasi experimental realizado con 37 participantes (Grupo de Intervención n = 20 y Grupo de Control n = 17). La intervención duró 8 meses. Los resultados de los cuidadores (sobrecarga y estrés) y sobrevivientes (grado de independencia) se midieron mediante las escalas de Zarit, Estrés Percibido y Katz, en los momentos: preintervención, cuarto mes de intervención y posintervención. Las diferencias entre estos resultados intergrupales e intragrupales y el tamaño del efecto se calcularon mediante las pruebas de Mann-Whitney y Friedman (ajuste de Bonferroni por Wilcoxon) y el coeficiente de Kendall W.

**Resultados:**

el Grupo de Intervención redujo la sobrecarga (p = 0,039) y el estrés (p = 0,009), principalmente después de ocho meses de intervención, lo que no se observó en el Grupo de Control. El grado de independencia no varió entre grupos o momentos (p>0,05). La intervención tuvo un tamaño de efecto moderado (p=0,45 y p=0,54).

**Conclusión:**

la intervención fue eficaz para reducir la sobrecarga y el estrés de los cuidadores familiares, pero no alteró el grado de independencia de los sobrevivientes de ACV, en comparación con el Grupo de Control.

## Introduction

Stroke is one of the main causes of chronic cognitive and functional impairments in adults and aged individuals worldwide. The rehabilitation of stroke survivors is generally long; the family, or a single family caregiver, assumes home care, with the execution of complex tasks and without due preparation, which results in physical, economic, emotional and psychosocial burden^(1)^.

In the absence of a source of social support, especially health services and health, this burden can increase and jeopardize both the health of the caregiver and the quality of care provided to the patient. This can result in the emergence or worsening of stress in the caregiver and in the difficulty recovering the autonomy of the stroke survivors to carry out the everyday basic activities^(2)^.

These negative consequences for the caregiverstroke survivor dyad show the need for reducing such burden. Thus, the literature recommends enabling to these caregivers multicomponent interventions that emphasize care for their own health, while providing continuous training for the care of the stroke survivor^(3)^.

Nurses can be at the forefront among the health professionals that are part of formal social support^(4)^, establishing partnerships with other members of the multidisciplinary team in the implementation of strategies or multicomponent interventions to provide support, education and advice to the caregivers, either individually or collectively.

These interventions must be developed considering the caregivers’ needs^(5)^ and the recovery phase of the stroke survivors, which is divided into acute phase (occurring between the transition period from hospital discharge to the first six months in the community) and the chronic phase (starting from six months after returning to the community)^(3)^.

Although the caregiver’s burden remains throughout the care period^(6)^, there are more publications of intervention studies in the acute phase, which reveals a need for more interventionist research studies in the chronic phase^(3)^, since the caregivers need constant guidelines on elementary and complex care, knowing that problems can emerge during the care period.

Most of the research studies on support interventions after stroke, focusing on the caregivers, come from highincome countries. In Brazil, the interventions to reduce the burden on the family caregiver family caregivers of survivors of this disease based on scientific evidence are scarce^(5)^, especially in the chronic phase of stroke. In this sense, interventions with low financial cost and easy development can be viable in health care, especially in the public sector, so as to supplement routine care or as an alternative to it, usually provided by the public and private health services.

As the burden generally has relation with the caregiver’s stress and with the stroke survivor’s independence level^(7-8)^, the following hypothesis will be tested: There will be differences in the burden and stress of family caregivers and in the independence level of stroke survivors in the Intervention Group, when compared to the Control Group. Therefore, the objective of the study was to analyze the effectiveness of a support intervention on the burden and stress of family caregivers and on the stroke survivors’ independence level compared to the Control Group.

## Method

### Study design

A prospective and quasi-experimental study, based on the Transparent Reporting of Evaluations with Nonrandomized Designs (TREND) statement, which represents a supplement to the Consolidated Standards of Reporting Trials (CONSORT) statement^(9)^.

Randomization is a process that distributes a “random” number of participants in different groups (experimental and control) in order to preserve the characteristics that similar between them. For a study to be classified as randomized, it is necessary that all the randomization stages (allocation sequence generation, concealment and implementation mechanism) are rigorously performed^(10)^.

Thus, this study was not classified as randomized because allocation sequence generation occurred by means of the only alternative and acceptable method called minimization, which, although not eliminating bias in all the known and unknown factors, allows for the balance between the Intervention and Control groups, by selecting the participants’ factors^(10)^. The choice of this method is recommended for smaller groups and was necessary due to the lethality of stroke, which could further reduce the sample.

The study was approved by the Research Ethics Committee of the State University of Southwest Bahia under CAAE 71341017.5.0000.0055, therefore observing Brazilian Resolution number 466 of 2012 and the Declaration of Helsinki. All the participants authorized the research by signing the Free and Informed Consent Form in two copies, one being filed by one of the researchers (first author) and the other remaining in possession of the caregivers.

### Participants and sample size

The sample was for convenience and the study took place in the city of Guanambi, Bahia (BA), Brazil. The family caregivers were recruited between September 2017 and March 2018, in their homes. Therefore, it was necessary to identify the stroke survivors in the chronic phase of the disease that had an attendance register from 2014, either in the regional hospital unit (n=152) or in the 17 Basic Health Units (BHUs) (n=69) managed by the Unified Health system (*Sistema* Único *de Saúde*, SUS).

The eligibility criteria for the caregivers were as follows: identifying themselves as the primary family caregivers of a stroke survivor who was in the chronic phase and presented care-related dependence in one or more functions (assessed by the Katz scale), time working as a caregiver between 6 and 50 months, being 18 years old or more, not receiving any financial remuneration for the care provided, living in urban areas and living in the same household as the stroke survivor or not.

### Intervention

The intervention consisted in supporting the family caregivers. Therefore, it was divided into two parts (individual and group) and lasted 8 months, combining the terms “acquisition of skills” and “education”.

Before starting the intervention, the lead researcher (first author) conducted a previous study that investigated the needs of all the family caregivers, regardless of their future allocations in the Intervention Group (IG) or Control Group (CG). The results showed that these caregivers needed education in health about stroke and about daily care to the survivors, having free time and getting assistance for their physical and mental health^(11)^. Knowing these needs supported the organization of the group and individual components of the intervention.

For preparing the group component, during the planning of the intervention, health professionals were selected using the following inclusion criteria: minimum experience of 1 year in caregiver and stroke survivor assistance and in higher education teaching in the areas of Medicine, Nursing, Psychology, Physiotherapy, Physical Education or Nutrition. To ensure adherence of these professionals to the study protocol, the lead researcher maintained regular contact according to the time availability of these employees, with a mean of three individual meetings (before starting the intervention) to elaborate the content of the themes and their respective informative booklets.

The execution of the group component of the support intervention took place in the auditorium of a public university for 8 months (one monthly session during two hours), with the participation of the previously selected health professionals and of the IG caregivers. The 8 sessions took place in the form of thematic conversation circles and were guided by Paulo Freire’s theoretical and methodological framework on the pedagogy of autonomy, adapted to the study context^(12)^.

An action plan was developed for all the sessions containing information relating to their respective stages: problematization (description of the problem to be discussed), topic, date, time, locus, objectives, methodological strategies (presentation of the proposal, video, dynamics, group discussion, etc.), food for the participants, human and material resources, evaluation of each session and references used for each theme. Each session was recorded with a video camera by a research assistant and the following themes were covered: 1) the importance of self-care; 2) general guidelines on stroke (concept, types, risk factors and referral in the health care network); 3) negative and positive aspects of care; 4) care with nutrition in the dyad; 5) stress coping mechanisms in the caregiver; 6) positioning of the stroke survivor and caregiver and ergonomic posture of the caregiver; 7) deterioration in the health of the dyad and death of the person being cared for and 8) body care and body hygiene after stroke. Sessions one, three, five and seven started with a 30-minute relaxation exercise led by a Physical Education professional and sequentially mediated by a Psychologist, while the other sessions were conducted, respectively, by graduates in Medicine, Nutrition, Physiotherapy and Nursing. An informative booklet was handed out at the end of each session with the content of the theme addressed.

In the individual component, Physiotherapy and Psychology consultations were conducted by health professionals working in the health laboratory of a University Center and who did not participate in the group component. A total of 10 Physiotherapy sessions were availed, as well as unlimited Psychological care sessions until the end of the intervention, although the frequency of this service was related to the individual health needs of each caregiver.

The family caregivers allocated in the CG and in the IG were instructed to maintain their routine care in the public and/or private health units and, therefore, should not interrupt the usual care link with these services available to them, after accepting to participate in the study. The Control Group did not receive care from any health professional who participated in the support intervention.

To maintain adherence to the study, all the caregivers underwent routine laboratory tests offered by the SUS before the intervention, namely: blood tests [blood count, total cholesterol and lipoproteins, triglycerides, glycaemia, glutamic oxaloacetic transaminase (GOT), glutamic pyruvic transaminase (GPT), gamma glutamyl transferase (Gamma GT), hormone thyroidstimulating (HTS), thyroxine free flowing blood (T4 free), urea, creatinine and uric acid]; urinalysis (Type I urinalysis for dosage of the Sediment Abnormal Elements) and stool parasitologic test. Subsequently, the lead researcher delivered the results to the caregivers, offered verbal guidelines based on the SUS protocols and referred them to the BHUs for follow-up. Transportation was paid for the IG caregivers who lived far from the locus of the group sessions and a telephone contact was made during the previous week and another the day before the intervention to confirm the date, time and place of the session.

### Instruments and outcomes

A form containing variables about the caregiver and the stroke survivor was developed for this study, based on previous surveys^(1-2,4,7-8)^. The primary outcome was the caregiver’s burden in relation to the care provided to the stroke survivor. To assess it, the Zarit Burden Interview scale was used, consisting in 22 items that assess health condition, psychological and financial situation, interpersonal relationships and social and personal life. The total score of the scale varied from 0 to 88, where the higher the score, the greater the burden. Although it was developed for caregivers of older adults and of people with dementia, this scale can be applied to caregivers of people with various mental and physical ailments. In Brazil, its internal consistency and validity were tested in caregivers of older adults with depression and the Cronbach’s alpha value was 0.87^(13)^.

The secondary outcomes included the family caregivers’ stress and the stroke survivor’s independence level. The perceived stress scale was proposed in 1983 and validated for Brazil in the aged population in 2007; it consists of 14 Likert-type questions with answer options ranging from 0 to 4. The scores of the sum of the points obtained in the questions vary from 0 to 56. Higher score indicate more stress in the last 30 days. The reliability of the scale was tested in individuals aged from18 to 70 years old in a population-based study in Brazil and presented a Cronbach’s alpha of 0.775^(14)^.

To identify the stroke survivor’s independence level, the Basic Activities of Daily Living scale was applied to the caregivers. This instrument assesses the functional independence of older adults and of other dependents in six respective functions: feeding, sphincter control, transfer, going to the bathroom, ability to dress and bathe. The score varies from 0 to 6 points, where one point is assigned to each “yes” answer, the individual being considered as follows: independent in all functions (zero score) or dependent in one to six functions. This scale was cross-culturally adapted Brazil in older adults and their caregivers, having its internal consistency tested and approved by Cronbach’s alpha values ranging from 0.80 to 0.92. Its validity was not assessed directly, but the Cronbach’s alpha coefficient levels provided empirical evidence of its validity^(15)^.

All four instruments were applied in the homes of the family caregivers. To verify the suitability of these instruments to the study, a pilot test was performed with 8 caregivers who were not part of the sample (n=37).

### Data collection schedule

Data collection was conducted in-person and for the three outcomes (burden and stress of the caregiver and independence level of the stroke survivor) the application of the instruments took place in three stages: pre-intervention (T0) (between September 2017 and March 2018); during the fourth month of the intervention (T1) and post-intervention (T2) (between December 2018 and February 2019). The lead researcher trained a specialist nurse for collecting these data, with the last application of the scales occurring up to 3 months after the intervention has been completed.

### Allocation and blinding

After collecting data at the pre-intervention moment, a statistician (external to the research) conducted the generation of the participants’ allocation sequence, through the minimization process according to the outcomes, the sociodemographic characteristics of the caregiver and the stroke survivor, seeking homogeneity of these variables in both groups. In this allocation process (minimization), the first individual was randomly allocated and, for each subsequent participant, the treatment allocation which minimized the imbalance of the factors selected from the groups was identified^(10)^.

Blinding (blinding of the participants’ allocation in the Control and Intervention groups) was possible only for the trained nurse who applied all the instruments and for the statistician who conducted the minimization process and data analysis, since the caregivers and professionals were aware of the intervention.

### Statistical analysis

Medians and interquartile range (IQR), absolute values and percentages were used to respectively describe the continuous and categorical variables related to the caregivers’ characteristics [age, gender, marital status, income (in this study, the minimum wage varied between R$ 937.00 and R$ 998.00 reais - Brazilian money -, from 2017 to 2019 and the value of the last year was considered, corresponding to the value of the US dollar in 2019, which was R$ 3.6570), occupation, schooling, kinship, living with the patient, family support, care time and hours, burden and stress] and of the stroke survivor (age, gender and independence level), regardless of their integration into the experimental design. The differences in the median and proportion of these variables between the Intervention and Control groups were analyzed by using the Mann-Whitney test and Fisher’s Chi-square or exact test, respectively.

To assess the differences of the outcome variables analyzed (burden and stress of the caregiver; independence level of the stroke survivor) between the groups (i.e., Control and Intervention) at the same time, the Mann-Whitney test was used. For the intragroup analysis at the different moment (T0, T1 and T2), the Friedman tests were used (with Bonferroni adjustment by Wilcoxon). Effect size calculation was performed for the comparisons between groups (i.e., comparisons of the differences between T0, T1 and T2), Kendall’s W coefficient being adopted as the effect size indicator. Kendall’s W coefficient assumes that values equal to or near to 0 indicate a negligible effect size and that values close or equal to 1 indicate large effect^(16)^. The Statistical Package for Social Sciences (SPSS) software, version 21.0, was used for all the statistical analyses, considering a significance level of 5%.

## Results

The results are presented according to the TREND statement. Of the 44 participants eligible for the study, 37 consented to participate, being assigned to the Intervention (n=20) and Control (n=17) groups.

**Table 1:** Descriptive variables of the caregivers (n=37), stroke survivors (n=37) and the respective primary and secondary outcomes, with equivalence between the Intervention (n=20) and Control (n=17) groups. Guanambi, BA, Brazil, 2017-2019

Variables	Intervention Group (n=20)	Control Group (n=17)	P
**Family caregiver**			
Age in years old*	51.50 (36.25 - 62.72)	46.00 (35.00 - 65.50)	0.156^†^
Gender^‡^			0.584^§^
Female	19 (95.0%)	15 (88.2%)	
Male	1 (5.0%)	2 (11.8%)	
*Marital status^‡^*			
With a partner	15 (75.0%)	11 (64.7%)	0.495^§^
No partner	5 (25.0%)	6 (35.3%)	
*Income^‡^ (minimum wages||)*			
Less than one	15 (75.0%)	10 (58.8%)	0.295^§^
Greater than or equal to one	5 (25.0%)	7 (41.2%)	
*Work activity (Occupation)^‡^*			
Works away from home	3 (15.0%)	4 (23.5%)	0.680^§^
Does not work	17 (85.0%)	13 (76.5%)	
*Schooling (years of study)^‡^*			
Less than 10	3 (15.0%)	2 (11.8%)	0.012^§^
Greater than or equal to 10	17 (85.0%)	15 (88.2%)	
*Kinship^‡^*			
Son/daughter and spouse	19 (95.0%)	15 (88.2%)	0.075^§^
Other	1 (5.0%)	2 (11.8%)	
Lives^‡^ with the stroke^¶^ survivor			0.647^§^
Yes	18 (90.0%)	16 (94.1%)	
No	2 (10.0%)	1 (5.9%)	
Care time (months)*	13.00 (6.00 -22.50)	14.00 (7.00 -32.00)	0.238^†^
Hours of care per week*	14.50 (8.75 - 11.00)	14.00 (11.00 - 19.00)	0.996^†^
*Family support ^‡^*			
Yes	13 (65.0%)	7 (41.2%)	0.147^§^
No	7 (35.0%)	10 (58.8%)	
**Stroke^¶^ survivor**			
Age in years old*	77.50 (63.00 - 85.00)	66.00 (53.00 - 81.50)	0.156^†^
*Gender^‡^*			
Female	9 (45.0%)	7 (41.2%)	0.815^§^
Male	11 (55.0%)	10 (58.8%)	
**Outcome measures**			
Caregiver burden*	31.50 (18.50 - 43.00)	25.00 (15.60 - 38.50)	0.776^†^
Caregiver stress*	27.00 (18.00 - 31.75)	24.00 (15.50 - 33.50)	0.714^†^
Independence level*	4.00 (2.50 - 5.00)	3.00 (3.00 - 5.00)	0.505^†^

* Continuous variables represented by median and interquartile range;

† P = Referring to the Mann-Whitney test, considering p<0.05;

‡ Categorical variables represented by number and percentage;

§ P = Considering p<0.05, the chi-squared test was used when the expected frequency in the table cells was greater than five, and Fisher's exact test when the expected frequency was less than five; ||Minimum wage (the minimum wage varied over the three years of the study - 2017 to 2019 - between R$ 937.00 and R$ 998.00, and the value of the last year was considered, which corresponds to the value of the US dollar in 2019, which was R$ 3.6570), Brazil, 2017-2019;

¶ Stroke = Cerebral Vascular Accident

**Figure 1:**
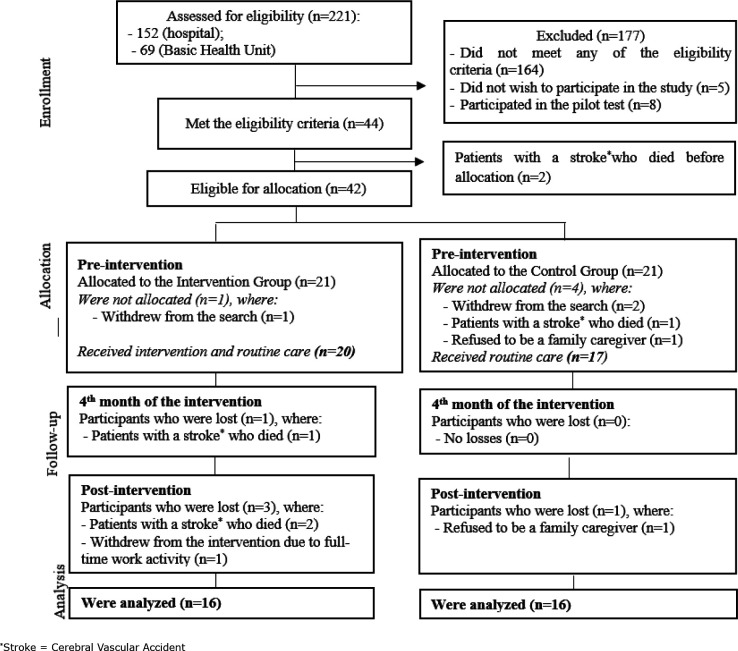
Flowchart of the study participants. Guanambi, BA, Brazil, 2017-2019

The baseline descriptive variables of the caregivers, stroke survivors and primary (caregiver burden) and secondary (caregiver’s stress and Stroke survivor’s independence level) outcomes in the Intervention and Control groups were tested for homogeneity, both groups being statistically similar. Most of the caregivers in the study were women, married, with low financial income, and unemployed. Of these, more than half lived in the same house that the stroke survivors, were their children or spouses and provided care several hours of the day, regardless of the stroke survivor’s gender. Despite the homogeneity of the samples, the IG presents a higher median age, schooling level, family support and stroke survivors with a greater degree of functional impairment, as shown in Table 1.

**Table 2:** Comparison between the scores of the primary and secondary outcomes in the Intervention (n=16) and Control (n=16) groups at the pre-intervention, during the intervention and post-intervention moments. Guanambi, BA, Brazil, 2017-2019

Outcome variables	T0* Median; IQR^§^	T1^†^ Median; IQR^§^	T2^‡^ Median; IQR^§^	P	Effect size
**Caregiver burden**					
IG^||^	33.50 (18.50-44.50)	25.50 (15.00-37.75)	25.00 (16.25-31.50)^¶^	0.039**	**0.45**
CG^††^	34.00 (25.25-39.25)	28.00 (17.50-35.50)	28.00 (23.00-42.75)	0.444	0.23
**Caregiver stress**					
IG^||^	29.00 (18.00-33.50)	22.00 (12.50-29.75)^‡‡^	15.50 (10.0-29.25)^¶^	0.009**	**0.54**
CG^††^	24.50 (17.50-33.75)	20.50 (16.25-26.75)	22.00 (13.00-27.00)	0.167	0.33
**Katz^§§^**					
IG^||^	4.00 (2.25-4.75)	4.00 (1.25-5.00)	4.00 (0.25-5.00)	0.542	0.20
CG^††^	3.00 (2.25-5.00)	4.00 (3.00-5.00)	4.00 (2.25-5.00)	0.584	0.18

* T0 = Pre-intervention;

† T1 = During the intervention (4th month);

‡ T2 = Post-intervention;

§ IQR = Interquartile range;

|| IG = Intervention Group;

¶ The post-intervention moment is different from the pre-intervention;

** P = p<0.05;

†† CG = Control Group;

‡‡ The moment corresponding to the intervention is different from the pre-intervention;

§§ Katz = Stroke survivor's independence level

Regarding the family caregivers’ adherence to the protocol of this study, it is noteworthy that all the patients underwent the laboratory tests. For the IG, the number of sessions in which the caregivers participated varied from 4 (n=3) to 6 or more (n=13) and, of these, only 7 people accepted transportation payment for the group component of the intervention. Of the 10 caregivers who required psychological care, 6 did not start the sessions because of the difficulty of scheduling by telephone contacts, one reported that the group service was sufficient to improve emotional support and three caregivers attended only 1-4 sessions, as they mentioned that the group meetings offered better psychological well-being. Of the 12 caregivers assessed for the physiotherapy sessions, 8 attended between 6 and 10 sessions and the absences were justified by: increased blood pressure levels (n=1) and lack of family support on the scheduled dates and times (n=3) (data not recorded in table).

The median burden, stress and independence level were not significantly different (p>0.05) in the Control and Intervention groups at any of the three moments. On the other hand, the moments assessed differed significantly for two caregiver variables studied in the IG. For the caregiver burden variable, a reduction in the median between the moments was evidenced, ranging from 33.50 at T0 to 25.00 at T2. For the caregiver stress variable, there was also a reduction in the median between the moments, mainly ranging from 29.00 at T0 to 15.50 at T2. On the contrary, the stroke survivor’s independence level did not present any difference between the moments, maintaining a constant median of 4.00. The results also point to a moderate effect size on the outcome variables of the caregiver, which shows the effectiveness of the support intervention over the proposed time, since the Intervention Group presented a difference over time, which was not the case in the Control Group (Table 2).

## Discussion

Considering the mean effect size found in this study, the results indicate that a support intervention, centered on the caregivers, can reduce the burden and stress from care, but still cannot improve the independence of stroke survivors in the chronic phase over time. The outcome variables analyzed are multifactorial and, therefore, if other interventions were added to this, they may result in an even greater positive effect for the participants.

Most of the other multicomponent interventions that provided social support to family caregivers occurred during the acute phase of stroke, as this period is marked by the apex of burden and stress from care. For these aforementioned studies, the main modality used was (psycho)educational, through the individual instrumentalization to the caregiver, about care practices and problem solving. However, these studies presented divergent results, with the significant reduction or not of caregiver burden and stress in the Intervention Group^(1,17)^, which suggests the importance of implementing and assessing these interventions over time.

On the other hand, multicomponent interventions conducted in the chronic phase of stroke, with the group modality, related to the educational and skills acquisition measures, showed a significant reduction in the caregivers’ burden and stress, in the only group evaluated. The main effects for the participants were as follows: better support among the caregivers, greater self-confidence and self-care, increased ability to cope with problems and management of negative feelings, incentive to using the community resources and applying knowledge about the care techniques^(18-19)^.

Unlike the aforementioned studies that were developed in the chronic phase of stroke, this support intervention used two groups (Intervention and Control), which reinforces the greater effect of the group strategy in reducing the caregivers’ burden and stress during home care, especially in the psychosocial aspects.

Generally, coexistence in a support group awakens the perception of not being alone and is preferable to individual care. Social support between the group members and the professionals improves the feeling of emotional well-being^(20)^, mainly in the sharing of successful experiences for problem solving. The participants of this study also made these findings explicit.

Knowing that other people were able to modify their care routine can motivate the caregivers to develop their own autonomy to acquire a new perspective towards the care of the other and self-care, which leads to a release of their distresses and anguishes. As a result, they can present reduced stress and burden.

The extension of the intervention of this study is in line with what the literature recommends, between 5 and 9 sessions^(3)^. In opposition to most of the research studies already published, the caregivers’ individual needs were incorporated. However, focusing this intervention on the caregiver may have little influence on the improvement of the stroke survivors’ results^(3)^, which was also evidenced in this research.

Similarly to this support intervention, another family-centered study, which used a short message service and cell phone calls for 8 weeks, with the objective of improving the basic daily activities of stroke survivors, also failed to increase the participants’ independence in the chronic phase of this disease^(21)^. It is probable that the focus of this support intervention, in responding only the unmet needs of the caregivers, influenced this result. Stroke survivors may also have reached the apex of their motor recovery prior to the study, as it is during the acute phase that the professionals intensify the use of therapies for musculoskeletal strengthening^(22)^ and, therefore, after hospital discharge, the survivors can present functional decline. In addition to that, the way in which the family caregivers stimulated the stroke survivors’ mobility and independence at home after receiving guidelines in the group component was not verified in this research. Verifying these care practices can ease understanding of the effect of the intervention on the independence of these survivors for the daily activities.

Most of the recovery of the motor functions occurs until the sixth month post-stroke and, after this period, functional capacity decreases progressively until it stabilizes^(23)^. Some factors can interfere with the recovery of these functions, such as the early use of antithrombotic therapy, type of stroke^(23)^, its severity at admission and advanced age of the survivors^(24)^, its severity at hospital admission and older age of survivors

The CG participants also maintained the stroke survivors’ independence level and reduced the caregivers’ burden and stress, although the results have not been significant. The performance of laboratory tests and the referral to the health unit may have influenced the CG caregivers in lifestyle change, greater concern for health and search for assistance support, which may have modified the results of this study.

These general data indicate that there is need for a continued intervention to the caregivers and their family members who have survived a stroke, so that there is an improvement in this dyad’s health and well-being over time. Those interventions can be preferably coordinated by nurses in their work spaces, adapting them to the needs of caregivers and patients during the individual home education strategies or in the training of the support groups in the community^(25)^ as light and low financial cost technologies.

Most of the interventions in health developed on the theme of stroke focus on support in the recovery and rehabilitation of survivors of this disease. Few interventions have been directed to the caregiver’s needs^(20)^, as well as to their effect towards the stroke survivors. In Brazil, this scenario is even more critical, since these interventions in health are still scarce. Therefore, this support intervention that has been developed represents an advance in scientific knowledge and contributes to the clinical practice, as it emerges as an alternative or complementary model to the current methods of daily care in the public or private health services for the reduction of the caregivers’ burden and stress. The greater adherence of the participants in the group component suggests the preference for this approach in relation to the individual component (psychological support). As a care manager and main executor of the health education actions in the various health units, nurses can be in charge of conducting that intervention, mainly in the use of the group component.

In intervention studies, it is not always possible to blind researchers and participants due to the interaction between them, which can generate some bias in the result. However, to reduce this risk, the evaluator and data collector were blinded and a Control Group was used, as recommended by the TREND statement, which represent strengths of this study. In addition to these points, the minimization process was adopted for the participants allocated in the Intervention and Control groups, since it was not possible to perform randomization.

The limitations of the study refer to the sample size and to the non-randomization of the sample, which precludes generalizing the results to other groups and, therefore, the data must be interpreted with caution. Therefore, in future research studies, a larger sample selected by randomization can be used, especially when analyzing sustainability over time. Even so, the findings in this study may help in the design of such research studies.

## Conclusion

The intervention was effective to directly reduce the family caregivers’ burden and stress in the post-stroke chronic phase. However, the repercussion of its effects on stroke survivors was not identified, as they did not present changes in their independence level for the performance of the basic daily activities, when compared to the Control Group.

Such findings reinforce that care to the caregivers is essential to improve their physical and psychological health and should not be limited to the early months of their role. In addition to assessing and incorporating the stroke survivors’ needs, in this intervention, may generate more benefits for these participants.
